# Transcriptional profiles of genes related to electrophysiological function in *Scn5a* ^+/−^ murine hearts

**DOI:** 10.14814/phy2.15043

**Published:** 2021-10-07

**Authors:** Michael Takla, Charlotte E. Edling, Kevin Zhang, Khalil Saadeh, Gary Tse, Samantha C. Salvage, Christopher L.‐H. Huang, Kamalan Jeevaratnam

**Affiliations:** ^1^ Faculty of Health and Medical Science University of Surrey Guildford UK; ^2^ Christ’s College University of Cambridge Cambridge UK; ^3^ School of Medicine Imperial College London London UK; ^4^ Clinical School University of Cambridge Cambridge UK; ^5^ Second Hospital of Tianjin Medical University Tianjin China; ^6^ Department of Biochemistry University of Cambridge Cambridge UK

**Keywords:** arrhythmia, Brugada syndrome, mechanisms, sodium channel, transcription

## Abstract

The *Scn5a* gene encodes the major pore‐forming Na_v_1.5 (α) subunit, of the voltage‐gated Na^+^ channel in cardiomyocytes. The key role of Na_v_1.5 in action potential initiation and propagation in both atria and ventricles predisposes organisms lacking *Scn5a* or carrying *Scn5a* mutations to cardiac arrhythmogenesis. Loss‐of‐function Na_v_1.5 genetic abnormalities account for many cases of the human arrhythmic disorder Brugada syndrome (BrS) and related conduction disorders. A murine model with a heterozygous *Scn5a* deletion recapitulates many electrophysiological phenotypes of BrS. This study examines the relationships between its *Scn5a* ^+/−^ genotype, resulting transcriptional changes, and the consequent phenotypic presentations of BrS. Of 62 selected protein‐coding genes related to cardiomyocyte electrophysiological or homeostatic function, concentrations of mRNA transcribed from 15 differed significantly from wild type (WT). Despite halving apparent ventricular *Scn5a* transcription heterozygous deletion did not significantly downregulate its atrial expression, raising possibilities of atria‐specific feedback mechanisms. Most of the remaining 14 genes whose expression differed significantly between WT and *Scn5a* ^+/−^ animals involved Ca^2+^ homeostasis specifically in atrial tissue, with no overlap with any ventricular changes. All statistically significant changes in expression were upregulations in the atria and downregulations in the ventricles. This investigation demonstrates the value of future experiments exploring for and clarifying links between transcriptional control of *Scn5a* and of genes whose protein products coordinate Ca^2+^ regulation and examining their possible roles in BrS.

## Graphical abstract

Volcano plot of differentially expressed pre‐selected genes, contrasting transcription in ventricular *Scn5a* ^+/−^ to wild type. The *y*‐axis indicates unadjusted *p* values based on Student’s *t*‐tests, while the *x*‐axis indicates the log_2_ of each normalized fold change.

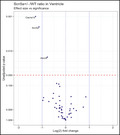

## 1 INTRODUCTION

The Brugada syndrome (BrS) poses a major worldwide public health problem, accounting for one in five sudden cardiac deaths among patients without reported structural cardiac defects (Antzelevitch et al., 2005; Matsuo et al., 2001). It is inherited as an incompletely penetrant autosomal dominant trait. Of clinical BrS cases, 10 to 30% have an identifiable causal mutation (Chen et al., 1998). Of these, the most frequent involve loss‐of‐function in the *Scn5a* gene (Chockalingam et al., 2012).

Clinical Type I BrS is characterized by electrocardiographic right precordial coved‐type ST elevations with a transient or stable lead V1–V3 T‐wave inversion (Gussak et al., 1999; Kurita et al., 2002), and significantly elevated risks of polymorphic ventricular tachycardia, atrial fibrillation, and ventricular fibrillation (Amin et al., 2010; Kusano et al., 2008). The nature of the relationship between ST elevation and tachyarrhythmia is uncertain. Early experiments in canine hearts had suggested a repolarization hypothesis, invoking acute decreases in inward Na^+^ current (*I*_Na_), during phase 1 of the right ventricular epicardial action potential resulting in regional differences in transmural repolarization (Yan & Antzelevitch, 1999). In contrast, subsequent experimental and some clinical studies suggested a depolarization hypothesis (Meregalli et al., 2005) in which a compromised *I*_Na_ slows the conduction velocity of the epicardial action potential, doing so to a greater extent in the right ventricular outflow tract than in its remaining myocardium (Nagase et al., 2002), predisposing to re‐entrant excitation (Morita et al., 2003).

Murine models permit investigations relating particular mutations to their phenotypic consequences. The *Scn5a* ^+/−^ mouse recapitulates some of the clinical‐ and age‐dependent features of BrS (Papadatos et al., 2002) and related proarrhythmic disorders, such as progressive cardiac conduction defect (Guzadhur et al., 2012; Probst et al., 2003; Tan et al., 2001) despite its differing myocardial and chamber volumes, heart rates, regional ion channel distributions (Zimmer et al., 2014), and ventricular action potential waveforms. The parallels extended to associations between Scn5a haploinsufficiency and age‐related fibrotic changes (Jeevaratnam et al., 2010; Nademanee et al., 2015) that may reflect noncanonical roles of voltage‐gated sodium channels in cardiac homeostasis (Abriel, 2010; McNair et al., 2004).

The BrS phenotype has been associated with close to 300 distinct genomic mutations (Kapplinger et al., 2010) though in most their causal relationships with the arrhythmic phenotype are not directly apparent (Hosseini et al., 2018). This abundance and diversity of disease‐causing variants suggests that that aging interacts with a polygenic, rather than Mendelian, background in producing BrS phenotypes. Previous studies had examined the age‐related factors in development of arrhythmic risk in *Scn5a* ^+/−^ murine hearts (Dautova et al., 2010), prolongation of PR and QRS intervals (Jeevaratnam et al., 2010; Royer et al., 2005), and their sex‐dependence, the latter revealing accentuated male over female phenotypes (Jeevaratnam et al., 2010). Fewer had examined the effects of *Scn5a* haploinsufficiency on non‐electrophysiological aspects of cardiomyocyte function. This study explores the possible contributions of transcriptional alterations involving genes potentially related to electrophysiological phenotypes following heterozygotic *Scn5a* deletion in a murine model.

## 2 MATERIALS AND METHODS

### 2.1 Animals

Replacement of the *Scn5a* gene’s second exon with an SA‐GFP‐PGK neomycin cassette produced heterozygous genotypes in five male and three female mice aged 11 ± 3 months, bred on a 129sv background, as described for the previously established BrS model (Papadatos et al., 2002). Mice were housed at a facility with a 12‐h light/dark cycle at 21℃ with access to sterile chow (RM3 Maintenance Diet; SDS), water, bedding, and environmental stimuli, in accordance with the Animals (Scientific Procedures) Act 1986, United Kingdom Home Office regulations. All procedures therefore also complied with the Guide for the Care and Use of Laboratory Animals, United States National Institutes of Health (NIH Publication No. 85‐23, revised 1996). Animals were sacrificed by cervical dislocation (Schedule 1, UK Home Office Regulations).

### 2.2 Tissue samples

Hearts from four wild type (WT) and four *Scn5a* ^+/−^ mice were obtained through ex vivo removal. The atria were excised from the ventricles prior to snap‐freezing and subsequent separate storage at −80℃.

### 2.3 RNA isolation

RNA was isolated using the Monarch RNA isolation kit (New England Biolabs). Ventricular tissue was weighed and chopped into small pieces from which 30 mg was taken to protection buffer and homogenized with a Stuart handheld homogenizer until smooth. For atria the entire tissue sample was used. The manufacturer’s protocol was followed including removal of genomic DNA. The resultant RNA quantity and quality were evaluated by Bioanalyzer analysis following the manufacturer’s protocol (Agilent RNA 6000 Nano Kit; Agilent Technologies) and all RNA samples exhibited RNA integrity number values >7.

### 2.4 cDNA preparation

cDNA was prepared with the aid of High‐Capacity cDNA Reverse Transcription Kit (Applied Biosystems) according to the manufacturer’s protocol. One microgram of RNA was used for each sample. The cDNA was tested with SYBR Green qPCR for efficient reverse transcription and lack of genomic DNA, as described previously (Edling et al., 2019).

### 2.5 TaqMan array assay

Thermo Fisher custom TaqMan array cards were used to examine the gene expression of selected genes as described in the Section 3. Fifty‐five genes were assayed in all 16 samples (4 samples/group), and an additional 7 genes (*Gja1*, *Gja5*, *Hcn2*, *Scn1b*, *Scn2b*, *Scn3b*, and *Scn4b*) were assayed in 12 samples (3 samples/group). All assays on the cards were present in triplicate and pre‐validated by Thermo Fisher. The cards were run on a Quant 7 cycler following the manufacturer’s protocol without modifications.

### 2.6 Data analysis and statistical testing

With the QuantStudio software threshold set at 0.2 fluorescence units and the baseline range automatically assigned, data were imported to Microsoft Excel for the application of the 2^−ΔΔCT^ method (Livak & Schmittgen, 2001). Determining the geometric mean of the Cq values of two housekeeping genes, *Actb* and *Gapdh*, made it possible to normalize and obtain fold changes from the transcript of each gene. Application of Student’s independent *t*‐tests on the processed data generated an estimate of the type I error rate, and, by extension, *p* value, of each change, for analysis of statistical significance.

## 3 RESULTS

**FIGURE 1:**
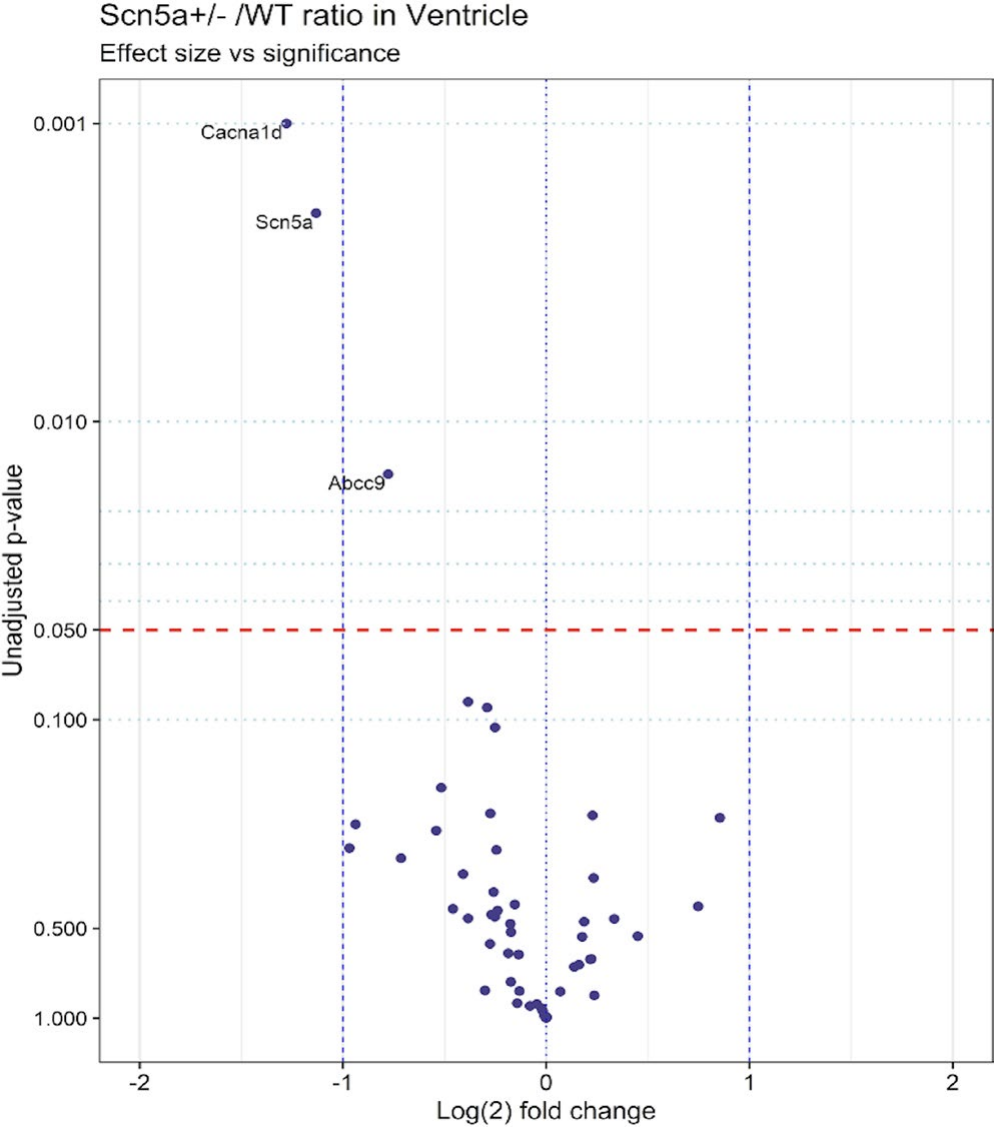
Volcano plot of differentially expressed pre‐selected genes, contrasting transcription in ventricular *Scn5a* ^+/−^ to wild type (WT). The *y*‐axis indicates unadjusted *p* values based on Student’s *t*‐tests, while the *x*‐axis indicates the log_2_ of each normalized fold change. Gene transcriptional with *p* values < 0.1 are labeled in the plot. The red line indicates significance level *p* = 0.05

**FIGURE 2:**
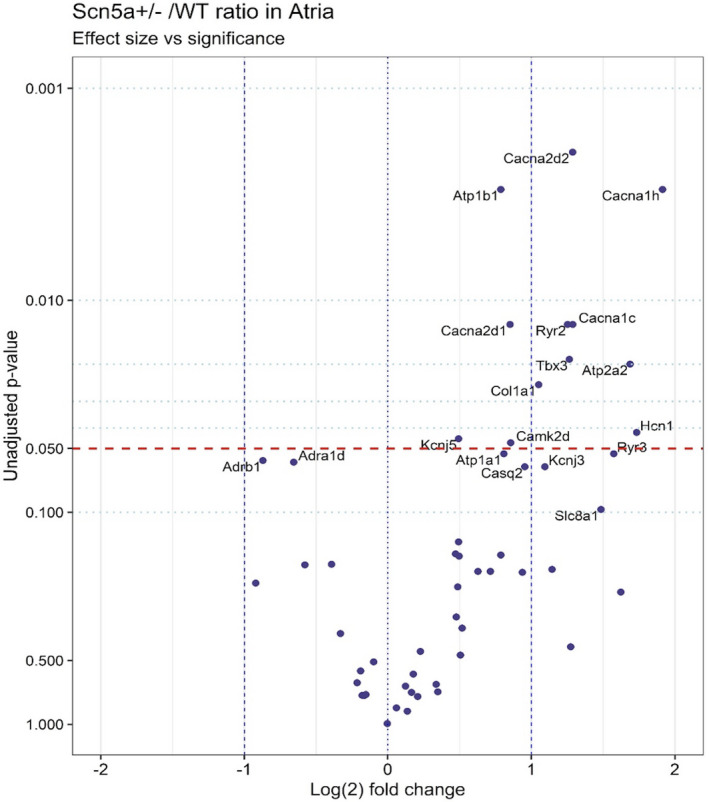
Volcano plot of differentially expressed pre‐selected genes, contrasting transcription in atrial *Scn5a* ^+/−^ to wild type (WT). The *y*‐axis indicates unadjusted *p* values based on Student’s *t*‐tests, while the *x*‐axis indicates the log_2_ of each normalized fold change. Gene transcriptional changes with *p* values < 0.1 are labeled in the plot. The red line indicates significance level *p* = 0.05

Of the 62 genes encoding systematically selected diverse cardiac electrophysiological or homeostatic functions (Huang, 2017), the Student’s *t*‐test to a *p* < 0.05 significance level of each normalized fold change, demonstrated significant changes in concentrations of mRNAs transcribed from 15 distinct genes. Of these, *Scn5a* expression was expectedly halved in ventricular, but was contrastingly not significantly downregulated in atrial tissue suggestive of feedback mechanisms increasing the expression of the WT allele. Of the 14 remaining genes showing an altered expression, none were shared by both atria and ventricles (Figures 1 and 2), with most exclusive to atrial tissue (Figure 2). Notably, of the statistically significant changes in gene expression, all those in the atria were upregulations, and all those in the ventricles were downregulations.

### 3.1 Na^+^/K^+^‐ATPase

The Na^+^/K^+^‐ATPase is a heterotrimeric protein. Its *Atp1a* (α) subunit is targeted to the plasma membrane (Geering, 2001) by the K^+^‐occluding (Lutsenko & Kaplan, 1993) *Atp1b* (β) subunit. Its activity in exporting three Na^+^ for every two K^+^ it imports (Post & Jolly, 1957) is modulated by the FXYD subunit (Garty & Karlish, 2006 ; Therien & Blostein, 2000). This contributes a minor intrinsic electrogenicity. However, it is the resulting K^+^ electrochemical gradient that maintains most of the negative resting membrane potential (*E*_m_), against its dissipation through expressed 2‐pore domain (K_2P_3.1; *Kcnk3*) channels (Ketchum et al., 1995). The murine *Scn5a* ^+/−^ atria showed significant, 111%, increase in *Atp1b1* mRNA levels and doubled though not significant (*p* = 0.053) *Atp1a1* levels relative to WT (Table 1).

**TABLE 1:** Heat map showing the statistical significance of changes in the expression of functional groups of genes tested by TaqMan array assay for transcriptional alterations in *Scn5a+/−*, relative to wild‐type, murine hearts. Mean expression value with wild type normalised to 1.0 and standard error of the mean (SEM) in italics. Yellow indicates no change, green indicates downregulation and red indicates upregulation of expression. Darker shades of red/green reflect larger magnitudes of fold changes in gene transcription

Functional group	Gene	Ventricles				Atria			
		WT		SCN5A^+/−^		WT		SCN5A^+/−^	
		Mean	±SEM	Mean	±SEM	Mean	±SEM	Mean	±SEM
Na^+^/K^+^‐ATPase activity	Atp1a1	1.0	*0.0*	0.8	*0.0*	1.0	*0.0*	2.2	*0.5*
	Atp1a2	1.0	*1.1*	0.9	*0.5*	1.0	*0.2*	1.2	*0.4*
	Atp1b1	1.0	*0.0*	1.2	*0.1*	1.0	*0.0*	2.1	*0.3*
Ion channels controlling resting membrane potential (P‐IV)	Abcc8	1.0	*0.2*	0.5	*0.0*	1.0	*0.1*	1.0	*0.1*
	Abcc9	1.0	*0.9*	0.6	*0.5*	1.0	*0.1*	1.2	*0.1*
	Kcnj3	1.0	*0.0*	1.2	*0.0*	1.0	*0.1*	2.1	*0.2*
	Kcnj5	1.0	*0.0*	0.8	*0.0*	1.0	*0.1*	1.4	*0.1*
	Kcnj8	1.0	*0.1*	0.9	*0.2*	1.0	*0.1*	0.9	*0.1*
	Kcnj11	1.0	*0.2*	0.7	*0.1*	1.0	*0.1*	0.9	*0.2*
	Kcnj12	1.0	*0.1*	0.8	*0.1*	1.0	*0.1*	1.1	*0.2*
	Kcnk3	1.0	*0.1*	1.0	*0.1*	1.0	*0.1*	1.0	*0.3*
Ion channels initiating excitation	Hcn1	1.0	*0.1*	1.8	*0.2*	1.0	*0.0*	3.3	*0.1*
	Hcn2	1.0	*0.1*	0.8	*0.1*	1.0	*0.1*	1.0	*0.1*
	Hcn4	1.0	*0.3*	0.6	*0.1*	1.0	*0.0*	3.1	*0.2*
Ion channels permitting *I*_Na_ (P‐0)	Scn5a	1.0	*0.1*	0.5	*0.1*	1.0	*0.1*	0.9	*0.1*
	Scn7a	1.0	*0.1*	0.9	*0.1*	1.0	*0.1*	1.7	*0.3*
Subunits modulating the kinetic profile of *I*_Na_	Scn1b	1.0	*0.4*	0.5	*0.1*	1.0	*0.2*	1.7	*0.4*
	Scn2b	1.0	*0.2*	1.1	*0.1*	1.0	*0.1*	1.2	*0.4*
	Scn3b	1.0	*0.3*	1.4	*0.2*	1.0	*0.3*	1.1	*0.1*
	Scn4b	1.0	*0.1*	2.6	*0.5*	1.0	*0.2*	1.4	*0.3*
Ion channels controlling repolarization (P‐III)	Kcna4	1.0	*0.3*	0.9	*0.1*	1.0	*0.0*	1.4	*0.4*
	Kcnd3	1.0	*0.1*	1.0	*0.1*	1.0	*0.0*	1.6	*0.2*
	Kcne1l	1.0	*6.2*	1.2	*4.3*	1.0	*1.1*	2.4	*7.3*
	Kcnh2	1.0	*0.1*	1.0	*0.1*	1.0	*0.1*	0.8	*0.2*
	Kcnn1	1.0	*0.1*	1.0	*0.1*	1.0	*0.0*	0.9	*0.1*
	Kcnn2	1.0	*0.1*	1.1	*0.2*	1.0	*0.5*	1.2	*0.2*
Ion channels permitting surface *I*_Ca_	Cacna1c	1.0	*0.4*	1.0	*0.5*	1.0	*0.0*	2.4	*0.1*
	Cacna1d	1.0	*0.0*	0.4	*0.0*	1.0	*0.2*	1.6	*0.2*
	Cacna1g	1.0	*0.1*	0.9	*0.2*	1.0	*0.1*	1.1	*0.2*
	Cacna1h	1.0	*0.0*	0.8	*0.0*	1.0	*0.0*	3.8	*0.1*
Subunits modulating surface *I*_Ca_ trafficking and kinetics	Cacnb2	1.0	*0.1*	0.9	*0.3*	1.0	*0.2*	1.9	*0.7*
	Cacna2d1	1.0	*0.1*	1.0	*0.1*	1.0	*0.0*	1.8	*0.1*
	Cacna2d2	1.0	*0.0*	0.7	*0.0*	1.0	*0.1*	2.4	*0.1*
Intracellular ion channels, transporters, and enzymes controlling Ca^2+^ homeostasis	Atp2a2	1.0	*0.5*	1.4	*0.6*	1.0	*0.1*	3.2	*0.3*
	Camk2d	1.0	*0.0*	0.9	*0.1*	1.0	*0.0*	1.8	*0.1*
	Casq2	1.0	*0.9*	0.5	*0.1*	1.0	*0.0*	1.9	*0.2*
	Ryr2	1.0	*0.1*	0.8	*0.1*	1.0	*0.0*	2.4	*0.1*
	Ryr3	1.0	*0.0*	1.0	*0.0*	1.0	*0.1*	3.0	*0.3*
	Slc8a1	1.0	*0.2*	1.3	*0.4*	1.0	*0.1*	2.8	*0.6*
Surface adrenergic receptors	Adra1a	1.0	*8.6*	0.8	*2.1*	1.0	*0.7*	0.5	*0.3*
	Adra1b	1.0	*0.1*	0.8	*0.0*	1.0	*0.1*	0.7	*0.1*
	Adra1d	1.0	*0.1*	1.0	*0.1*	1.0	*0.1*	0.6	*0.1*
	Adrb1	1.0	*0.1*	0.8	*0.1*	1.0	*0.1*	0.6	*0.2*
	Adrb2	1.0	*0.6*	0.8	*1.0*	1.0	*0.3*	1.3	*1.5*
Proteins, and their subunits, involved in the adenylyl cyclase pathway	Adcy4	1.0	*0.1*	1.2	*0.1*	1.0	*0.1*	1.4	*0.1*
	Adcy5	1.0	*0.1*	1.2	*0.0*	1.0	*0.1*	1.4	*0.2*
	Pde2a	1.0	*0.1*	1.0	*0.1*	1.0	*0.1*	0.9	*0.1*
	Pde4d	1.0	*0.1*	0.9	*0.1*	1.0	*0.1*	1.4	*0.3*
	Prkaca	1.0	*0.2*	0.8	*0.1*	1.0	*0.1*	0.8	*0.2*
	Prka1a	1.0	*0.1*	1.1	*0.1*	1.0	*0.1*	1.4	*0.1*
	Prka2a	1.0	*0.1*	0.9	*0.2*	1.0	*0.1*	1.4	*0.1*
	Prka2b	1.0	*0.0*	1.7	*0.2*	1.0	*0.1*	1.1	*0.3*
Fibrotic markers	Col1a1	1.0	*0.1*	1.1	*0.1*	1.0	*0.1*	2.1	*0.2*
	Col3a1	1.0	*0.3*	0.8	*0.2*	1.0	*0.1*	2.2	*0.6*
	Tgfb1	1.0	*0.1*	1.1	*0.1*	1.0	*0.0*	1.1	*0.2*
Gap junction connexins	Gja1	1.0	*0.2*	0.7	*0.2*	1.0	*0.2*	2.0	*0.2*
	Gja5	1.0	*0.2*	0.9	*0.2*	1.0	*0.3*	1.2	*0.2*
	Gjd3	1.0	*0.6*	0.8	*0.4*	1.0	*0.1*	1.3	*0.8*
Other genes	Tbx3	1.0	*0.2*	1.1	*0.4*	1.0	*0.1*	2.4	*0.2*
	Trpc1	1.0	*0.1*	0.7	*0.1*	1.0	*0.1*	1.4	*0.1*

SEM, standard error of mean; WT, wild type.

### 3.2 Ion channels controlling *E*_m_

In phase 4 of the atrial or ventricular cardiomyocyte action potential, *E*_m_ is stabilized to approximately −90 mV. Although the electrochemical gradients required to maintain *E*_m_ depend on Na^+^/K^+^‐ATPase activity, it is the inwardly rectifying K^+^ channels (Kir) making the major contribution to its steady‐state value. Of these, the resulting inwardly rectifying I_K1_ conductance permits K^+^ influx, and prevents K^+^ efflux when *E*_m_ is more negative and positive, respectively, than its resting value. The underlying regionally heterogeneous tetrameric (Wang et al., 1998) Kir2.1 (Kcnj2), Kir2.2 (Kcnj12), and Kir2.3 (Kcnj4) owe their inward rectification to intracellular polyamines (Ficker et al., 1994; Lopatin et al., 1994), and, to a lesser extent, Mg^2+^ (Matsuda et al., 1987; Vandenberg, 1987).

The hetero‐octameric sarcolemmal and mitochondrial ATP‐sensitive K^+^ channel (K_ATP_) makes a smaller, nevertheless still significant, contribution to *I*_K1_. ATP binding to the four Kir6.2 (*Kcnj11*), and to a lesser extent to the Kir6.1 (*Kcnj8*) subunits (Seino, 1999) reduces its open probability. In contrast, ATP binding to the four SUR2A (*Abcc9*), or especially in the atria, SUR1 (*Abcc8*) subunits (Seino & Miki, 2004) facilitates its own hydrolysis to ADP, activating the channel in the presence of Mg^2+^. The dependence of such a mechanism of inward rectification on [ATP]_i_ enables ischemic preconditioning in the face of transitory periods of ischemia (O’Rourke, 2000). Finally, GIRK4 (*Kcnj5*) constitutes one of the most modulable fractions of the resting *I*_K1_. Binding of vagally released ACh to G_i_‐linked M_2_AChRs expressed by the sinoatrial node (SAN) promotes the dissociation of its component βγ complex and α subunit of the trimeric G protein. This increases the open probability of the GIRK4 pore hyperpolarizes resting *E*_m_ and so reduces heart rate (Wickman et al., 1998).

Murine *Scn5a* ^+/−^ ventricles, but not their atria showed significant, 42% reductions in *Abcc9* mRNA levels relative to WT, but no other significant differences in transcription of genes controlling *E*_m_. In contrast, *Scn5a* ^+/−^ atria showed significant increases in *Kcnj5* transcription levels and also a trend (*p* = 0.061) toward markedly higher *Kcnj3* level relative to WT (Table 1).

### 3.3 Ion channels initiating excitation

Cardiac pacemaker cell automaticity depends at least in part on tetrameric hyperpolarization‐activated cyclic nucleotide‐gated (HCN) channels (Brown & Difrancesco, 1980). The membrane hyperpolarization following the preceding action potential removes the auto‐inhibitory effect of the HCN voltage‐sensitive domain increasing its pore open probability, actions further enhanced by cAMP binding (Flynn & Zagotta, 2018). A voltage clock component of pacing activity results from the balance between its consequent inward *I*_f_ current with a coincident K^+^ efflux resulting in membrane depolarization. In addition, a Ca^2+^ clock driven by depolarizing sarcolemmal Na^+^/Ca^2+^ exchange current (*I*_NCX_) stimulated by the resultant rhythmic sarcoplasmic reticular (SR) release of Ca^2+^ (Bogdanov et al., 2001) contributes to the regulation of the pacemaker frequency. The resulting SAN excitation is propagated successively to the atrioventricular node (AVN), bundle of His and Purkinje fibers, and then to the ventricular cardiomyocytes.

*Hcn4* is the predominant SAN Hcn isoform (Baruscotti et al., 2011) and atrioventricular bundle; *Hcn1* and *Hcn2* are also selectively expressed by the AVN and bundle branches (Herrmann et al., 2011). Despite lower global expression levels (Günther & Baumann, 2015), *Hcn3* also contributes to shaping ventricular cardiomyocyte action potential waveforms (Fenske et al., 2011). We observed little detectable alteration in expression of the *Hcn3* gene in either atria or ventricles. *Scn5a* ^+/−^ and WT ventricles showed no significant differences in *Hcn1*, *Hcn2*, or *Hcn4* mRNA concentrations. In contrast, *Scn5a* ^+/−^ atria showed increased (by 233%) transcription of *Hcn1* mRNA compared to WT (Table 1).

### 3.4 Ion channels involved in *I*_Na_

The steeper depolarizing, phase 0, of atrial and ventricular cardiomyocyte action potential compared to that of pacemaker cells, reflects their expression of voltage‐gated Na^+^ (Na_v_) channels. The pore‐forming (α) subunit of the predominant Na_v_1.5 (*Scn5a*) cardiac isoform, and possibly voltage sensing Na_v_2.1 (*Scn7a*) (Hiyama et al., 2002) comprises four domains (DI–DIV), each with six helical segments (S1–S6). Cardiomyocyte depolarization causes a repulsion of basic Arg and Lys residues located at every third position of S4 (Schwartz & Stühmer, 1984) driving an outward rotation that activates a rapid first‐order activation. The predominant component of the subsequent inactivation proceeds through a fast (2–10 ms) ball and chain interaction mediated by the IFM motif of the DIII–DIV loop (Goldin, 2003). The cytoskeleton‐anchoring auxiliary (Isom, 2001 ; Malhotra et al., 2000, 2002) *Scn1b*–*Scn4b* (β) subunits modulate the subsequent voltage‐dependent return of Na_v_1.5 from its inactivated to its resting conformation. We here report no significant differences between *Scn5a* ^+/−^ and WT hearts in the levels of expression of these genes (Table 1), except *Scn5a* itself in the ventricles.

### 3.5 Ion channels mediating action potential repolarization

The early rapid phase 1 and delayed phase 3 action potential repolarization phases that follow phase 0 depolarization and the phase 2 plateau, respectively, are driven by particular voltage‐gated K^+^ (K_v_) channel subtypes. Their K_v_ α subunits form pseudo‐tetramers that show a similar steady‐state voltage sensitivity of activation as, but markedly differing kinetics from, those of Na_v_. These require all four α subunits to undergo a simultaneous, even if cooperative, shifts from closed to intermediate to open conformations. There are marked heterogeneity between isoforms. The relatively rapidly activating K_v_1.4 (*Kcna4*), K_v_4.2 (*Kcnd2*), and K_v_4.3 (*Kcnd3*) mediate the early phase 1 A‐type currents *I*_to1,s_ and *I*_to1,f_, respectively (Angelo et al., 2002). K_v_7.1 (*Kcnq1*) mediates the slowly activating phase 3 delayed‐rectifier current (*I*_Ks_), whose voltage‐dependence is modulated by co‐assembled KCNE5 (*Kcne1l*) (Angelo et al., 2002). The K_v_11.1 (*Kcnh2*) (Vandenberg et al., 2012) and K_v_1.5 (*Kcna5*) (Feng et al., 1997) activates in successfully shorter timeframes to result in the rapid (*I*_Kr_), and ultra‐rapid atrial (*I*_Kur_) currents, respectively. Furthermore, in the atria (Skibsbye et al., 2014), the small conductance Ca^2+^‐activated K^+^ (SK) channels, K_Ca_2.1 (*Kcnn1*) and K_Ca_2.2 (*Kcnn2*) (Tuteja et al., 2005), further contribute to repolarization.

There were no significant differences in the concentrations of mRNA transcribed from any of their evaluated genes responsible for the repolarization between *Scn5a* ^+/−^ and WT mice in either ventricular or atrial tissue (Table 1).

### 3.6 Ion channels involved in *I*_Ca_

The phase 2 plateau phases can typically extend over 300 ms. Here K^+^ efflux arising from activation of *I*_Ks_, *I*_Kr_, and *I*_Kur_ is balanced by Ca^2+^ influx mediated by sarcolemmal voltage‐gated Ca^2+^ current. The predominant isoform of pore‐forming (α) subunit of the underlying voltage‐gated Ca^2+^ (Ca_v_) channels, Ca_v_1.2 (*Cacna1c*) and Ca_v_1.3 (*Cacna1d*), expressed to a lesser extent shows strong homologies with Na_v_1.5 (Tanabe et al., 1988). It generates a surface L‐type Ca^2+^ current (*I*_Ca‐L_) with a distinct, very slow, inactivation. Furthermore, the α subunit complexes with both *Cacnb2 (β*) and γ and *Cacna2d1*/*2* (α2γ) subunits. α2γ enhances β‐mediated (Pragnell et al., 1994) channel trafficking (Felix et al., 1997) in addition to modulating the kinetic profile of I_Ca‐L_. In contrast, neither Ca_v_3.1 (*Cacna1g*) nor Ca_v_3.2 (*Cacna1h*) require accessory subunits for expression and permission of surface T‐type Ca^2+^ current (*I*_Ca‐T_).

In *Scn5a* ^+/−^ ventricles, *Cacna1d* transcript levels showed a significant decrease of 59% compared to WT, with no further significant changes in mRNA levels corresponding to the remaining genes above. *Scn5a* ^+/−^ atria showed significant increases, >80% in *Cacna1c*, *Cacna1h*, *Cacna2d1*, and *Cacna2d2* expression (Table 1).

### 3.7 Intracellular ion channels, transporters, and enzymes controlling Ca^2+^ homeostasis

In addition to surface Ca^2+^ fluxes, intracellular Ca^2+^ homeostasis involves Ca^2+^ movements between SR and cytosol. SR Ca^2+^–release channel ryanodine receptors (Ryr) activate upon binding of the cytosolic Ca^2+^ derived from the initial *I*_Ca_, inducing a Ca^2+^‐induced Ca^2+^ release that couples excitation to cardiomyocyte contraction (Fabiato, 1983). Ryr2 is the most common cardiac isoform, though Ryr3 (Perez et al., 2005) is also expressed. SERCA (Atp2a2) mediates active Ca2+ transport into the SR; Ca2+ binding to calsequestrin (Casq2) then reduces free [Ca2+]SR (Knollmann, 2009), facilitating SR membrane Ca2+ transport. Along with triadin (Caswell et al., 1991) and junctin (Jones et al., 1995), Casq2 also complexes with Ryrs (Zhang et al., 1997). Ca^2+^/CaM‐dependent kinase II (Camk2d) regulates Ca^2+^ homeostasis at all points along this axis (Rodriguez et al., 2003; Wehrens et al., 2004), as well as phosphorylating, and modulating the behavior of, several Na_v_ subtypes (Burel et al., 2017), K_v_ (Li et al., 2007; Tessier et al., 1999; Wagner et al., 2009), and Ca_v_ (Blaich et al., 2010) channels.

*Scn5a* ^+/−^ ventricles showed no significant changes in concentrations of mRNAs transcribed from the above relative to WT. In contrast, *Scn5a* ^+/−^ atria displayed significant *Atp2a2*, *Ryr2*, and *Camk2d* upregulation, with increases in expression of 222%, 138%, and 81%, respectively, as well as a trend (*p* < 0.1) toward greater apparent *Ryr3* and *Casq2* transcription (Table 1).

### 3.8 Surface adrenoceptors

Surface adrenoceptor (AR) activation further modulates cardiomyocyte action potential waveforms. Aside from the G_q_‐linked α_1_‐AR (*Adra1a*; subtype A), neither *Adra1b* nor *Adra1d* (subtypes B and D) elicit differential net negative and positive inotropic effects in either the right or left ventricles (Wang et al., 2006). Cardiomyocytes express G_s_‐linked β‐ARs. β_1_‐ARs (*Adrb1*) predominate, but Ca_v_1.2‐coupled β_2_‐ARs (*Adrb2*) also contribute to the generation of positive chronotropy, inotropy, and lusitropy, through mechanisms including G_i_‐mediated phospholipase A_2_ activation (Pavoine & Defer, 2005).

However, *Scn5a* ^+/−^ showed no significant atrial or ventricular differences in levels of mRNA transcribed from genes encoding these surface adrenoceptors from WT (Table 1).

### 3.9 Proteins, and their subunits, involved in the adenylyl cyclase pathway

The adenylyl cyclase pathway, exemplified by β‐AR signaling, mediates changes in chronotropy, inotropy, and lusitropy. Its central hub is the tetrameric enzyme phosphokinase A (Pearce et al., 2010). Of its two catalytic subunits, *Prkaca* encodes one subtype, and two autoinhibitory regulatory subunits––subdivided into types I, for example, *Prkar1a*, and II, for example, *Prkar2a* or *Prkar2b*. It is colocalized via A‐kinase anchoring proteins (AKAPs) (Bauman & Scott, 2002) with phosphodiesterases (PDEs), including PDE2 (*Pde2a*) and PDE4 (*Pde4d*). This establishes a high cAMP turnover rate. Large fold changes in [cAMP]_i_ relieve the allosteric autoinhibition of catalytic subunits, such that the intact holoenzyme (Smith et al., 2017) can initiate a phosphorylation cascade.

Neither *Scn5a* ^+/−^ atria nor ventricles showed any significant differences in the concentration of RNA transcribed from genes encoding proteins, and their subunits, involved in the adenylyl cyclase pathway relative to WT.

### 3.10 Fibrotic markers

Fibrosis involves replacement of myocardial with connective tissue resulting in the remodeling of cardiac chambers (Travers et al., 2016). It is driven by transforming growth factor‐β (TGF‐β) (Walton et al., 2017) activating canonical and non‐canonical pathways culminating in myofibroblast stimulation. This increases the formation relative to degradation of extracellular matrix. The resulting deposition of types I (*Col1a1*) and III (*Col3a1*) collagen fibrils creates scar tissue (Khalil et al., 2017).

Neither *Scn5a* ^+/−^ atria nor *Scn5a* ^+/−^ ventricles showed significant differences in *Tgfb1* expression compared to WT. In contrast, *Scn5a* ^+/−^ atria showed a significant upregulation of *Col1a1* mRNA by 107% relative to WT (Table 1).

### 3.11 Gap junction connexins

The component cardiomyocytes of each cardiac chamber create an electrical syncytium through formation of gap junctions, each consisting of two hemichannels. The connexon is a hexamer of connexin subunits, of which Cx43 (*Gja1*) and Cx40 (*Gja5*) are the most important for ventricular (Verheule et al., 1997) and atrial propagation of excitation (Gollob et al., 2006), respectively. Although murine Cx30.2 occasionally heteromizes (Gemel et al., 2008) with such connexins in the AVN, expression of the human orthologue Cx31.9 (*Gjd3*) protein may be undetectable in the myocardium (Kreuzberg et al., 2009).

cDNA corresponding to *Gdj3* was reliably detected. However, there were no significant differences in the levels of the gap junction connexin mRNAs between *Scn5a* ^+/−^ and WT mice, in either atria or ventricles (Table 1).

### 3.12 Other genes

T‐box transcription factor 3 (*Tbx3*) and transient receptor potential canonical 1 (*Trpc1*) are prerequisites for specifying the atrioventricular conduction system (Bakker et al., 2008) and for governing the hypertrophic response in failing cardiomyocytes (Seth et al., 2009), respectively.

*Scn5a* ^+/−^ ventricles showed no significant changes in *Tbx3* and *Trpc1* mRNA levels relative to WT mice. In contrast, *Scn5a* ^+/−^ atria exhibited significant upregulations of the former, with a 140% increase (Table 1).

## 4 DISCUSSION

This study examines the effects of diminished expression of Na_v_1.5 on the transcriptome of murine atrial and ventricular cardiomyocytes, and, in turn, the extent to which such significant changes reflect or explain electrophysiological observations. It surveys transcriptional changes in *Scn5a* ^+/−^ murine hearts hitherto used as an experimental model for BrS and related clinical conditions. Over 90% of the identified genomic mutations in BrS patients involve *Scn5a* (Chen et al., 1998). However, BrS has also been correlated with mutations in an additional 20 genes (Watanabe et al., 2011). These include genes related to ion channels controlling resting *E*_m_, such as Abcc9 (Hu et al., 2014) and Kcnj8; ion channels initiating excitation, such as Hcn4; subunits modulating *I*_Na_ kinetics, such as Scn1b–3b (Hu et al., 2009; Riuró et al., 2013; Watanabe et al., 2008); ion channels controlling repolarization, such as *Kcne3* (Delpón et al., 2008), *Kcne5*, and *Kcnd3*; and surface *I*_Ca_, such as *Cacna1c*, *Cacna2b* (Cordeiro et al., 2009), and *Cacna2d1*. Such findings are consistent with a multigenic backdrop in BrS, that could potentially involve evolution of its phenotype in the aged organism (Antzelevitch et al., 2005).

This study accordingly explores the effects of Na_v_1.5 haploinsufficiency on the transcriptome of atrial and ventricular cardiomyocytes, with a particular focus on the latter additional genes. Atria and ventricles showed differing transcriptional alterations arising from the *Scn5a* ^+/−^ genotype. The ventricular changes were limited to falls in the transcription of two unrelated genes: *Abcc9* and *Cacna1d*. The atrial *Scn5a* ^+/−^ genotype resulted in significant upregulations of 12 genes. Most of these changes clustered in the functional gene group regulating intracellular Ca^2+^ homeostasis, either involving surface currents (*Cacna1c*, *Cacna1h*, *Cacna2d1*, and *Cacna2d2*) or cytosolic and SR proteins (*Atp2a2*, *Camk2d*, and *Ryr2*). In addition, Na_v_1.5 haploinsufficiency may increase atrial predisposition to fibrosis via *Col1a1*.

Here we relate the present findings to previously reported electrophysiological features of *Scn5a* ^+/−^ murine hearts. First, the reduced ventricular Nav1.5 but normal or increased HCN expression reported here correlate with particular ventricular electrophysiological properties reported on earlier occasions. Previous physiological studies had reported that *Scn5a* ^+/−^ murine hearts were mildly bradycardic and showed increased risks of SAN block with age, and attributed these to altered *I*_Na_ rather than *I*_f_ (Lei et al., 2005). This study correspondingly reported that *Scn5a*+/‐ hearts showed unaltered ventricular *Hcn1*, *Hcn2*, or *Hcn4* expression and actual increases in atrial *Hcn1* expression compared to WT mice. In addition, young *Scn5a* ^+/−^ hearts showed impaired atrial, AV, and ventricular conduction velocity (Papadatos et al., 2002) consistent with the reduced overall Na_v_1.5 mRNA reported previously (Leoni et al., 2010) and 54% reduced ventricular Na_v_1.5 mRNA reported here. *Scn5a* ^+/−^ also showed accentuated QT dispersions, shortened ventricular action potential durations particularly involving the right ventricle recapitulating clinical observations (Ikeda, 2001) (Pitzalis et al., 2003), that may form the bases of both ST elevation and arrhythmia in BrS (Yan & Antzelevitch, 1999). These changes took place in an absence of altered expression in genes encoding ion channels controlling cardiomyocyte repolarization; the latter invite future investigations of increased right than left ventricular *Kcnd2* and *Kcnd3* expression and I_to_ density to similar extents in both Scn5a^+/−^ and WT hearts (Martin et al., 2012).

Second, the present findings show relatively normal atrial Na_v_1.5 mRNA expression, inviting future studies investigating for regionally specific negative feedback loop regulating atrial *Scn5a* transcription. This would then parallel previous reports describing differing levels of right and left ventricular *Scn5a* transcription in both WT and mutant mice hearts (Martin et al., 2012). In contrast, *Scn5a*+/‐ atria particularly demonstrated altered mRNA levels of gene products involved in surface Ca^2+^ current function such as *Cacna1c*, *Cacna1h*, *Cacna2d1*, and *Cacna2d2*, and of intracellular proteins, such as *Camk2d* and *Ryr2*, involved in [Ca^2+^]_i_ homeostasis with potential actions on *I*_Na_ (Table 1). In particular, the atrial upregulation of *Camk2d*, in Scn5a^+/−^ atria could over activate CaMKII known to phosphorylate Ser1933 and Ser1944 in the C‐terminal domain of Na_v_1.5, interfering with Ca^2+^/CaM‐directed alteration of its inactivation kinetics (Burel et al., 2017), as well as independently hyperpolarizing its steady‐state inactivation curve (Shah et al., 2006 ; Yoon et al., 2009). Furthermore, by phosphorylating Ser571 in its first intracellular loop, CaMKII augments the late *I*_Na_ (*I*_Na,L_) (Glynn et al., 2015). Similar events have been reported with excessive angiotensin II (Omar Velez Rueda et al., 2012) and reactive oxygen species (He & Zuo, 2015) in overactivating cardiomyocyte CaMKII. CaMKII also alters phosphorylation of targets such as RyR2 (Tian, 2004), the protein product of the *Ryr2* gene, itself also upregulated in *Scn5a* ^+/−^ atria. Moreover, gain‐of‐function mutations in *Ryr2* greatly reduce the protein expression of Na_v_1.5 in both atria (King et al., 2013) and ventricles (Ning et al., 2016), implying raised [Ca^2+^]_i_‐mediated acute and chronic Na_v_ inhibition.

**FIGURE 3:**
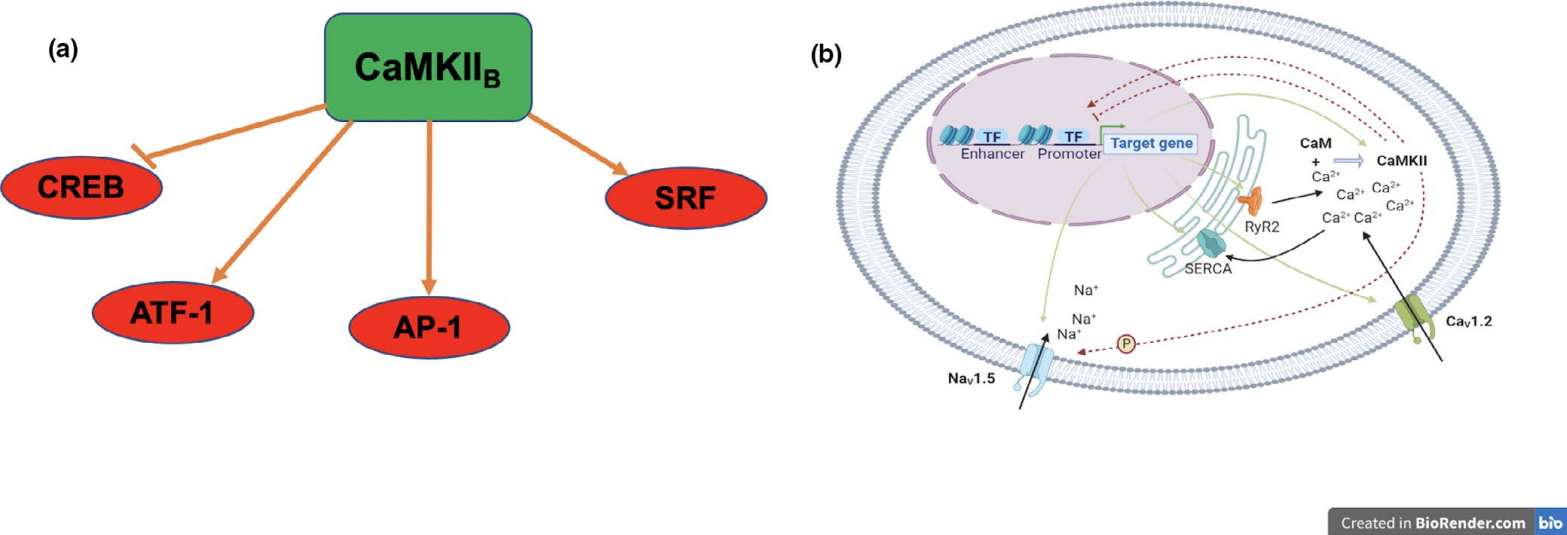
Through phosphorylation, Ca^2+^/CaM‐activated kinase II (CaMKII) introduces post‐translational modifications that influence the activities of various transcription factors (a). We posit a possible role for CaMKII, which post‐translationally modulates Na_v_1.5, in mediating the negative feedback loop and/or changes in the transcription of genes controlling cytosolic [Ca^2+^] (b) in the atria of *Scn5a* ^+/−^ mice. AP‐1, activator protein 1; ATF‐1, cAMP‐dependent transcription factor 1; CREB, cAMP response element‐binding protein; SRF, serum response factor. “Subscript B” refer to CaMKII isoforms

Such a relationship between changes in Na_v_1.5 function and Ca^2+^ homeostasis may involve post‐translational and/or transcriptional mechanisms. The CaMKIIδ_B_ isoform possesses a nuclear localization signal, providing a basis for its role in excitation‐transcription coupling (Figure 3a). Differential stimulation of nuclear CaMKII_B_ influences a range of transcription factors, including CREB (Sun et al., 1994), ATF‐1 (Shimomura et al., 1996), AP‐1 (Antoine et al., 1996), and SRF (Flück et al., 2000); the last is crucial in both initiating and maintaining the pre‐established cardiac transcriptional profile. This raises the intriguing possibility that CaMKII provides the missing link between the apparent negative feedback loop regulating atrial *Scn5a* transcription, and the upregulation of genes regulating intracellular Ca^2+^ homeostasis (Figure 3b).

Third, we here report that although *Scn5a* ^+/−^ atria and ventricles showed similar connexin expression levels and similar TGF‐β expression, *Scn5a* ^+/−^ atria showed a significantly upregulated *Col1a1* transcription relative to WT. This would parallel their interstitial fibrotic phenotype (Coronel et al., 2005 ; Frustaci et al., 2005) exacerbated by aging (Jeevaratnam et al., 2016).

The precise mechanistic links between the *Scn5a* ^+/−^ genotype, cardiac chamber‐specific transcriptional changes, and the BrS phenotype remains to be fully elucidated. However, this study reveals a clear link between Na_v_1.5 expression and calcium homeostasis in the atrial cardiomyocytes, with *Camk2d* a (speculative, but) possible mediator (Figure 3b). Moreover, the absence of gene expression alterations in the ventricular tissue might suggest that the level of Na_v_1.5 is not as critical as in the atria and that the compensatory mechanisms are either not activated or required.

Nonetheless, this tentative hypothesis must be regarded with caution. First, to avoid undermining the statistical power of this study, the relatively small sample of *Scn5a* ^+/−^ hearts was not divided into distinct groups of left and right atria and ventricles. Yet the lack of distinction made between the different sides of the hearts’ chambers may have obscured a potential laterality in gene expression, which, especially in light of functional data demonstrating left/right differences in the electrophysiology of BrS patient hearts (Pitzalis et al., 2003), merits future investigation. Second, and relatedly, to best simulate the effects of age‐related structural, molecular, and electrophysiological changes in human hearts on BrS risk and onset (Jeevaratnam et al., 2010; Nademanee et al., 2015; Papadatos et al., 2002), the *Scn5a* ^+/−^ hearts studies were exclusively those of aged mice, though future study could also add younger *Scn5a* ^+/−^ hearts as a control group. However, given similar sex‐related effects on BrS risk and *Scn5a* ^+/−^ murine heart function (Jeevaratnam et al., 2010), the lack of distinction made between the sexes (to avoid underpowering the study) likewise may have obscured sex‐specific changes in atrial or ventricular gene expression, again meriting further investigation. Finally, future study may benefit from western blotting to both verify whether translation reflects transcriptional changes, and to provide mechanistic insights into, for instance, changes in protein trafficking with *Scn5a* heterozygosity and differences therein between left and right atria and ventricles.

Despite these limitations, this study of cardiomyocyte transcriptional profiles in a murine *Scn5a* ^+/−^ model provides a path for further investigation into the molecular mechanisms underlying common arrhythmic disorders.

## CONFLICT OF INTEREST

None to declare.

## AUTHOR CONTRIBUTIONS

Michael Takla, Charlotte E. Edling, Kevin Zhang, and Samantha C. Salvage undertook the lab experiments, quality control, and technical troubleshooting; Michael Takla, Charlotte E. Edling, Khalil Saadeh, and Gary Tse undertook the data analysis; Michael Takla, Charlotte E. Edling, and Khalil Saadeh wrote the first draft of the manuscript. Christopher L.‐H. Huang and Kamalan Jeevaratnam designed the study, provided supervision, and secured funding for the work. All authors reviewed all subsequent drafts of the manuscript.
